# Biovalorisation of agro-industrial wastes into astaxanthin by *Xanthophyllomyces dendrorhous*

**DOI:** 10.1007/s00253-024-13257-5

**Published:** 2024-07-27

**Authors:** Osman N. Kanwugu, Ibrahim Ibn-Wuni, Vadim A. Shevyrin, Thomas C. Williams, Tatiana V. Glukhareva

**Affiliations:** 1 Institute of Chemical Engineering, Ural Federal University Named After the First President of Russia B.N. Yeltsin, Mira Street 28, 620002 Yekaterinburg, Russia; 2 School of Natural Sciences, and ARC Centre of Excellence in Synthetic Biology, Macquarie University, Sydney, NSW 2109 Australia

**Keywords:** Agro-industrial by-products, Bioconversion, Ketocarotenoid, *P. rhodozyma*, Soy molasses, Sustainability

## Abstract

**Abstract:**

Astaxanthin is a red xanthophyll with high economic and industrial value in the pharmaceutical, nutraceutical, cosmetic and food industries. In recent years, the biotechnological production of astaxanthin has attracted much attention as a sustainable alternative to the predominating petrochemical-dependent chemical synthesis. In this regard, *Xanthophyllomyces dendrorhous* is regarded as a promising microorganism for industrial production of astaxanthin. Unfortunately, biotechnological production of the carotenoid is currently expensive. The present study investigated soy molasses (SM) and residual brewers’ yeast as cheap fermentation feedstocks for the cultivation of *X. dendrorhous* and astaxanthin production. Yeast extract was obtained from residual brewers’ yeast using various techniques and then combined with SM to formulate a two-component growth medium which was subsequently used to cultivate *X. dendrorhous*. Generally, the yeast extract produced from residual brewers’ yeast supported *X. dendrorhous* growth and astaxanthin production at levels comparable to those seen with commercial yeast extract. Overall, cultivating *X. dendrorhous* in an SM-based medium containing 5% SM and 0.2% yeast extract obtained from residual brewers’ yeast resulted in significantly higher (> 20% more) biomass accumulation compared to the control media (YPD). A similar slightly higher astaxanthin output (up to 14% more) was recorded in the SM-based medium compared to YPD. The formulated cultivation medium in this study provides an opportunity to reduce the production cost of astaxanthin from *X. dendrorhous* while simultaneously reducing the environmental impact related to the disposal of the industrial waste used as feedstock.

**Key points:**

• *Cheap culture media were formulated from soy molasses and brewers’ spent yeast*

• *The formulated medium resulted in at least 20% more biomass than the control*

• *Up to 14% more astaxanthin was produced in molasses-based medium*

**Graphical Abstract:**

**Figure:**
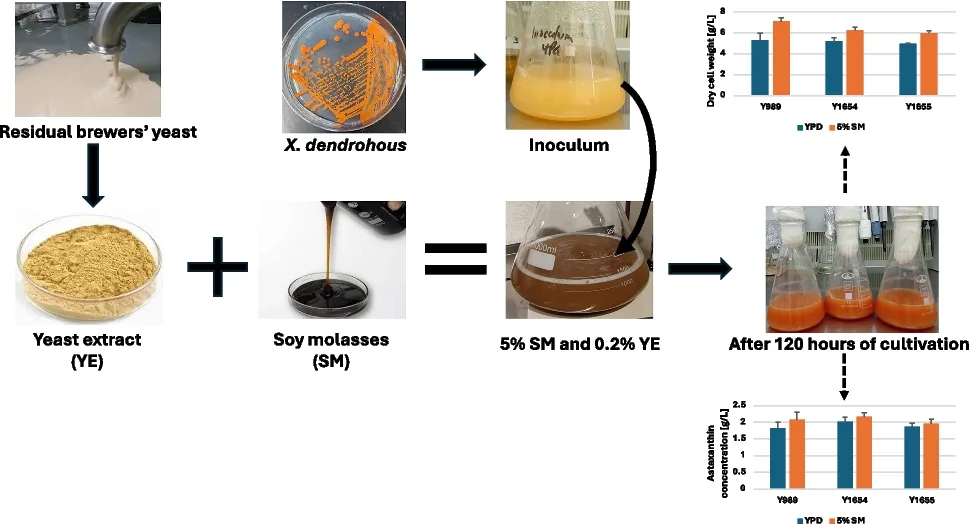

**Supplementary Information:**

The online version contains supplementary material available at 10.1007/s00253-024-13257-5.

## Introduction

Astaxanthin (3,3′-dihydroxy-4,4′-diketo-β,β-carotene) is an economically and industrially valuable carotenoid produced predominantly by microorganisms such *Haematococcus pluvialis*, *Xanthophyllomyces dendrorhous* (*Phaffia rhodozyma*) and *Paracoccus carotinifaciens*. It is a red–orange pigment responsible for the characteristic red–orange hues of the flesh of salmonids and exoskeleton and muscular epithelium of crustaceans such as shrimps, prawns, lobsters and krill (Ho and Shirakawa 2023; Kanwugu et al. 2022). Traditionally, consumers have linked this characteristic colour to product quality which as a result dictates, to some extent, the market value of these products. In addition, there is a growing interest in using astaxanthin to improve the pigmentation of poultry meat and eggs. Besides pigmentation, it has been widely reported that astaxanthin supplementation improves the overall health of farmed animals (Kanwugu et al. 2021; Ritu et al. 2023). Unfortunately, animals cannot synthesise astaxanthin de novo. Consequently, astaxanthin is a high-value feed additive in aquaculture and poultry farming. Nearly half of the global market for astaxanthin in 2023 is attributed to the aquaculture and feed industries (Grand View Research Inc. 2023). Additionally, astaxanthin has been shown to exhibit numerous biological activities; particularly famous is its superior antioxidant activity. It also possesses cardio- and neuro-protective effects, anti-diabetic and anti-cancer activities, immune-modulation and anti-inflammatory and anti-lipid peroxidation activities (Kanwugu et al. 2022; Liu et al. 2023). These have made astaxanthin appealing as a promising agent in human health and nutrition. As such, it is of high demand as a nutraceutical. Also, due to these potent biological activities, astaxanthin has attracted great attention in the food industry for developing functional foods (Stachowiak and Szulc 2021). In addition, it has anti-aging properties and offers protection against UV irradiation, making it appealing in the cosmetics industry as well (Davinelli et al. 2018).

At present, aquaculture remains the largest consumer of astaxanthin (Grand View Research Inc. 2023). The astaxanthin used in this sector is largely produced by chemical synthesis and is composed of all three stereoisomeric forms of astaxanthin: (3S, 3'S), (3R, 3'S)/(3S, 3'R) and (3R, 3'R). Over recent years, there has been a growing demand for astaxanthin in the food, nutraceutical and cosmetic industries (Nutakor et al. 2022). However, the lack of stereo-purity coupled with the rising concerns of synthetic additives makes chemically synthesised astaxanthin unattractive for direct human consumption (Stachowiak and Szulc 2021; Zhu et al. 2022). Accordingly, more effort is now being put into producing astaxanthin from natural sources. However, biotechnological production of astaxanthin, in general, is more expensive than chemical synthesis. While it costs around US$ 1000 to produce 1 kg of synthetic astaxanthin, it costs two to seven times as much to produce the same quantity of natural astaxanthin (Stachowiak and Szulc 2021). Currently, the freshwater microalgae *Haematococcus pluvialis* is the principal commercial source of natural astaxanthin, with a capacity to synthesise astaxanthin up to 5% of dry biomass. The astaxanthin produced by *H. pluvialis* is mainly the (3S, 3'S) astaxanthin isomer, the predominating isomer in nature. Besides *H. pluvialis*, *Xanthophyllomyces dendrorhous* is another important source of natural astaxanthin. This yeast produces principally the (3R, 3'R) isomer as its major carotenoid (Kanwugu et al. 2022; Liu et al. 2023). Even though the astaxanthin yield of *X. dendrorhous* (typically < 0.05% of DCW for wild-type strains) is, generally, lower than *H. pluvialis*, the yeast is regarded highly among industrial players as a very promising candidate for biotechnological production of astaxanthin (Kanwugu et al. 2020; Mussagy et al. 2021). This is because *X. dendrorhous* has easier cultivation conditions plus it is able to utilise different carbon and nitrogen substrates including wastes from the food and agriculture industry. It has been shown that astaxanthin productivity of the yeast can be increased (up to approximately 10 mg/g of DCW) with strain improvements and bioprocess optimisation (Gassel et al. 2013). Furthermore, it does not grow photosynthetically and can therefore reach high biomass densities. For instance, more than 80 g/L dry biomass has been obtained in bioreactor with sufficient mass transfer and aeration (de la Fuente et al. 2010; Gassel et al. 2013). This presents an opportunity to lower the production cost of natural astaxanthin by cultivating *X. dendrorhous* in low-cost, agri-food waste-based nutrient media (Kanwugu et al. 2020).

As a consequence of industrialisation and the ever-growing global population, gigatonnes of organic waste are generated yearly worldwide. At present, most of these wastes are either incinerated, dumped in landfills or used in composting. Disposal of agri-food wastes via these methods has severe environmental consequences including greenhouse gas emissions, contamination of waterways and the unsustainable use of resources such as soil nutrients and water (Arancon et al. 2013). It is therefore pertinent to find sustainable approaches to dealing with these wastes. In this regard, the United Nations highlights substantial reduction of waste and effective and environmentally friendly management of generated waste as a target for one of its Sustainable Development Goals (United Nations 2015). To improve the sustainability of the food chain, valorisation of agri-food wastes and by-products has become a focus in both research and the agri-food industry. Residual brewers’ yeast is the second largest by-product from the brewing industry and constitutes a major problem of the industry due to the large quantities generated almost daily. It is rich in carbohydrates, proteins, amino acids, lipids, minerals and vitamins, and as such, it is largely used in animal feed. Of late, it has become an attractive source of bioactive substances especially β-glucan and polyphenols (León-González et al. 2018; Puligundla et al. 2020). Due to its significant content of proteins, vitamins and minerals, it is equally appealing as a component of nutrient media for biotechnological application (Zarei et al. 2016). Similarly, soy molasses (SM) is a by-product of the soy processing industry, obtained after aqueous alcohol soy protein concentrate production. It contains carbohydrates (including glucose, fructose, galactose, sucrose, stachyose and raffinose) and proteins as well as minerals and vitamins. To date, soy molasses is almost exclusively used as an inexpensive animal feed component (Rakita et al. 2021). However, due to the presence of considerable amounts of fermentable sugars, nitrogenous compounds and some minerals, it has attracted attention in the field of biotechnology as a fermentation substrate.

This study investigated soy molasses and residual brewers’ yeast as cheap fermentation media components to cultivate *X. dendrorhous* for astaxanthin production. Yeast extract was obtained from residual brewers’ yeast and combined with soy molasses to formulate a growth medium which was subsequently used to cultivate *X. dendrorhous*.

## Materials and methods

### Feedstock

Residual brewers’ yeast was obtained from United Breweries Heineken LLC, Yekaterinburg Russia; soy molasses was obtained from Agroproduct JSC, Kaliningrad, Russia.

### Chemicals

Astaxanthin (synthetic) was purchased from ACROS, USA; Folin’s reagent was sourced from SIGMA, USA; agar was purchased from BIOLOT LLC, Russia; yeast extract was supplied by KATROSA REACTIV LLC, Russia; peptone and glucose were purchased from ORMET LLC, Russia; bovine serum albumin was supplied by DIAEM LLC, Russia; potassium hydroxide and sulphuric acid were supplied by CHIMREACTIV LLC, Russia; sodium hydroxide, copper sulphate and sodium potassium tartrate were supplied by CHEMREACTIVSNAB JSC, Russia; ethanol was supplied by ROSBIO, Russia; other solvents were procured from EKOS-1 Inc., Russia. All solvents were of analytical grade. Acetonitrile (LC–MS grade from Panreac, Barcelona, Spain) and water obtained using an ‘Aqualab AL-2 Plus’ (Mediana-Filter, Russia) water purification system were used for HPLC-HRMS analysis.

### Microorganisms and cultivation conditions

Strains of *X. dendrorhous* (*P. rhodozyma* Y989, Y1654 and Y1655), purchased from the Russian National Collection of Industrial Microorganisms, Moscow, were maintained on YPD growth medium (containing per litre: 20 g glucose, 10 g peptone, 2 g yeast extract and 9 g agar) by regular subculturing at 18 ℃ and then preserved at 4 ℃. For liquid cultures, yeasts were cultivated at 18 ℃ under continuous illumination of white light and constant agitation at 150 rpm. All seed cultures were prepared by cultivating yeasts in YPD broth for 48 h. For subsequent experiments, fresh cultivation media (YPD or molasses-based medium) were inoculated with seed culture at 10%.

### Preparation of yeast extract

Residual yeast was separated from leftover beer by centrifugation at 4470 × g for 10 min. The cell pellet was washed several times with distilled water until the supernatant after centrifugation was clear. Two percent aqueous cell suspension was prepared with 40 g of wet residual yeast biomass. Three parallel 5-mL aliquots were taken for DCW determination. Cell suspension was then subjected to one of three cell disruption methods as elaborated below. For each method, the supernatant was collected by centrifugation (4470 × g for 10 min), evaporated to dryness over water bath at 65 ℃ and stored at 4 ℃ for further experiments. The total protein content, as well as the elemental composition of the resultant yeast extracts, was characterised.

1. Autolysis: The pH of the suspension was adjusted to 6 and apportioned into 13 cotton-plugged conical flasks (250-mL capacity) and then incubated for 24 h at 50 ℃ with constant mixing at 150 rpm. The suspension was then pasteurised at 80 ℃ for 30 min and cooled to room temperature (Tanguler and Erten 2008).
2. Pressurised thermolysis: The suspension was apportioned into four conical flasks (1-L capacity) and autoclaved at 115 ℃ for 10 min. The autoclaved suspension was then cooled to room temperature (Zarei et al. 2016).
3. Ultrasonication: The suspension was placed in an ultrasonic bath (Elmasonic P 60 H) and subjected to ultrasonication (80 kHz, 100% power, 30–50 ℃) for 30 min, 200 mL at a time. The resultant was cooled to room temperature.

### Effect of different concentrations of soy molasses on *X. dendrorhous *growth and astaxanthin production

To identify the optimum amount of soy molasses (SM) for the growth of *X. dendrorhous* and astaxanthin production, different concentrations of soy molasses (3–10%) in the final media were evaluated. The desired amount of soy molasses was accurately weighed into a cotton-plugged conical flask and diluted with 400 mL of water. In a separate cotton-plugged conical flask, 1 g of yeast extract was dissolved in 100 mL of water. The two solutions are autoclaved and mixed under sterile conditions to constitute the molasses-based media. The final concentration of yeast extract in the resulting media was 0.2%. Where necessary, sterile water is added to compensate for lost volume during sterilisation. No peptone was added (in our previous article (Kanwugu et al. 2020), we observed a low astaxanthin production when soy molasses was supplemented with yeast extract and peptone. We speculated that the addition of peptone to the already nitrogen-rich soy molasses resulted in a reduced C/N ratio which negatively affected astaxanthin production). Growth media (100 mL each) were then inoculated with seed culture at 10% and then incubated at 12 ℃ for 120 h under continuous white illumination and agitation (150 rpm). At the end of the cultivation period, the concentration of residual sugar, dry cell weight (DCW) and concentration of astaxanthin were determined. For comparison, a similar experiment was carried out in parallel with a glucose-based medium (YPD) containing 2% glucose, 1% peptone and 0.2% yeast extract. All experiments were conducted in triplicates. The starting pH of the SM-based media ranged from 6.1 to 5.9, generally decreasing with increasing soy molasses concentration, while the pH of the control medium (YPD) was around 6.5.

### Evaluation of different yeast extracts in SM-based cultivation medium for *X. dendrorhous *growth and astaxanthin production

SM-based media containing 5% SM and 0.2% yeast (obtained by A, autolysis; B, pressurised thermolysis; or C, ultrasonication) were prepared as described in the previous section. Similar cultivation medium was prepared with a commercial yeast extract (S) as control. Each cultivation medium (in triplicate) was inoculated at 10% v/v with seed culture and incubated at 12 ℃ with continuous white light illumination and agitation (150 rpm) for 120 h. Residual sugar, concentration of astaxanthin and DCW were determined at the end of the cultivation period.

### Validating the ability of SM-based medium to support growth and astaxanthin production with different strains of *X. dendrorhous*

The ability of the proposed two-component medium (containing 5% SM and 0.2% yeast extract obtained from residual brewers’ yeast) was validated with other strains of *X. dendrorhous*. The cultivation medium was prepared as described previously with SM and yeast extract obtained via autolysis. The resultant medium was inoculated separately with seed cultures of *X. dendrorhous* strain Y989, Y1654 and Y1655. For each strain, the experiment was conducted in triplicate. Cultures were cultivated under the same conditions as described in the previous section for 120 h. The amount of residual sugar and the accumulation of astaxanthin and biomass were quantified at the end of the 120-h duration.

### Analytical methods

#### Protein quantification

The protein content of yeast extract was determined according to the Lowry method. Reagent A was composed of 2% Na_2_CO_3_ in 0.1 N NaOH. Regent B was made up of 0.5% CuSO_4_·5H_2_O in 1% potassium sodium tartrate tetrahydrate. Reagent C was composed of a fresh mixture of reagents A and B in a ratio of 50:1. An aliquot of yeast extract was dissolved in distilled water and then diluted to a concentration of 0.05 mg/mL. One millilitre of the yeast extract solution was mixed with 10 mL of reagent C and mixed gently for 10 min on a vortex. Folin’s reagent (0.5 mL) was added, and the resultant was incubated in the dark at room temperature for 30 min. The absorbance was immediately measured at 750 nm on a spectrophotometer (Shimadzu 1800-UV). The protein content was then estimated using a calibration curve prepared with bovine serum albumin (*R*^2^ = 0.993) (Lowry et al. 1951).

#### Elemental analysis

The carbon, nitrogen and hydrogen composition of the yeast extract was analysed with a PerkinElmer 2400 Series II CHNS/O analyser.

#### Quantification of residual sugar

The residual sugar at the end of the cultivation period was determined by the phenol sulphuric acid method as described in our previous paper (Kanwugu et al. 2020). The supernatant of culture broth was diluted with distilled water to 2 mL in a glass test tube; 50 µL of 80% aqueous phenol solution was added and then mixed thoroughly. Five millilitres of concentrated sulphuric acid was added and then incubated for 10 min in the dark and cooled down for 10 min in a 25 ℃ water bath. The absorbance was measured at 487 nm with a Shimadzu 1800-UV/Visible spectrophotometer. The amount of sugar was quantified with a glucose calibration curve (*R*^2^ = 0.997).

#### High-performance liquid chromatography-high resolution mass spectrometry (HPLC-HRMS) with tandem mass spectrometric (MS/MS) analysis

Characterisation of saccharides in soy molasses and culture supernatant was performed using an Agilent 1290 Infinity II HPLC system coupled with a tandem quadrupole time-of-flight (Q-TOF) mass spectrometer, the Agilent 6545 Q-TOF LC–MS (Agilent Technologies, USA). Chromatographic separation was carried out on a Luna Omega SUGAR 2.1 mm × 150 mm × 3.0 μm column (00F-4775-AN, Phenomenex) with an additional guard column. A solution of acetonitrile in water (75:25 v/v) was used as the mobile phase in isocratic mode. The column thermostat was maintained at 25 °C, and the flow rate was set at 0.35 mL/min. The total analysis time was 16 min. The Q-TOF instrument was operated with an electrospray ion source in negative ion mode. Nitrogen at 350 °C with a flow rate of 10 L/min was used as the drying gas, while the sheath gas temperature was set at 400 °C with a flow rate of 12 L/min. The nebuliser pressure was 40 psi, the capillary voltage was 3500 V, the skimmer voltage was 45 V and the fragmentor voltage was 90 V. Ions were scanned in the mass ranges of 100–1700 Da in MS mode with an acquisition rate of 3 spectra/s, and 30–800 Da in MS/MS mode. In MS/MS mode, the quadrupole was adjusted to isolate precursor ions with a bandwidth of Δ*m*/*z* 1.3. Collision-induced dissociation (CID) spectra of the precursor ions were obtained with collision energies (CE) of 20 and 30 eV. The collision cell was filled with nitrogen (99.999%). The *m*/*z* values in spectra were recorded with a measurement error of no more than 5 ppm. Adjustment and operation of the instrument, as well as data processing, were controlled by MassHunter Workstation Software B.08.00.

Tentative identification of the saccharides was performed by comparing their CID spectra, obtained from MS/MS experiments, with the spectra of carbohydrates from the NIST 2020 MS/MS Library.

#### Estimation of dry cell weight

Cells from 5 mL of culture broth were harvested in a pre-weighed falcon tube by centrifugation (4470 × g for 10 min). The cell pellet was washed twice with distilled water and then dried at 50 ℃ in an oven until a constant weight was reached. The weight difference was estimated as the DCW.

#### Extraction and quantification of astaxanthin

Cells from 10-mL culture broth were harvested and washed twice with distilled water. Astaxanthin was extracted and quantified as described previously (Kanwugu et al. 2020) with slight modifications. The cell pellet was resuspended in 2 mL of DMSO and lysed by ultrasonication (Bandelin Sonorex Digitec DT 31 H) for 15 min. The cell lysate was extracted repeatedly with petroleum ether until the extract was colourless. The petroleum ether layers were pooled together, and excess water was removed with anhydrous sodium sulphate. Sodium sulphate was filtered out and rinsed several times with fresh petroleum ether. The solvent was then evaporated on a rotary evaporator at 35 ℃. The residue was redissolved in 1-mL acetonitrile. Astaxanthin was quantified on a spectrophotometer at 478 nm using a linear astaxanthin calibration curve made with chemically synthesised astaxanthin (*R*^2^ = 0.993). HPLC characterisation of extracts was carried out as described previously (Kanwugu et al. 2020). Accordingly, we evaluated the effect of different concentrations of soy molasses on astaxanthin production in *X. dendrorhous* strain Y1655. Preliminary characterisation with HPLC–UV/Vis revealed that astaxanthin content accounted for ≥ 80% of the total carotenoids produced in all the strains tested in this study (Figure S1 [Supplementary information]). Therefore, we measured and presented the total carotenoid content as an equivalent of the astaxanthin content throughout this study for convenience.

## Results

### Characterisation of soy molasses and yeast extract

To ensure an adequate supply of the essential components such as carbon and nitrogen in the cultivation medium, it is necessary to characterise the rather heterogeneous feedstock. The concentration of total sugar in the soy molasses (SM) used for this study was determined to be 350.5 ± 1.7 mg/g, which amounts to approximately 35% w/w of sugar in the molasses. Further characterisation revealed that sucrose, raffinose and stachyose are the main sugars in the soy molasses, accounting for up to 80% of the total area of all detected peaks. Individually, sucrose is the most abundant, followed by stachyose. However, the combined proportion of raffinose and stachyose accounted for approximately 43% of the total area of all detected peaks (Figure S3 [Supplementary Information]). The elemental analysis also revealed that SM contains an appreciable amount of nitrogen-containing compounds: carbon (C) = 26.70 ± 2.14% and nitrogen (N) = 1.95 ± 0.03%.

**Fig. 1:**
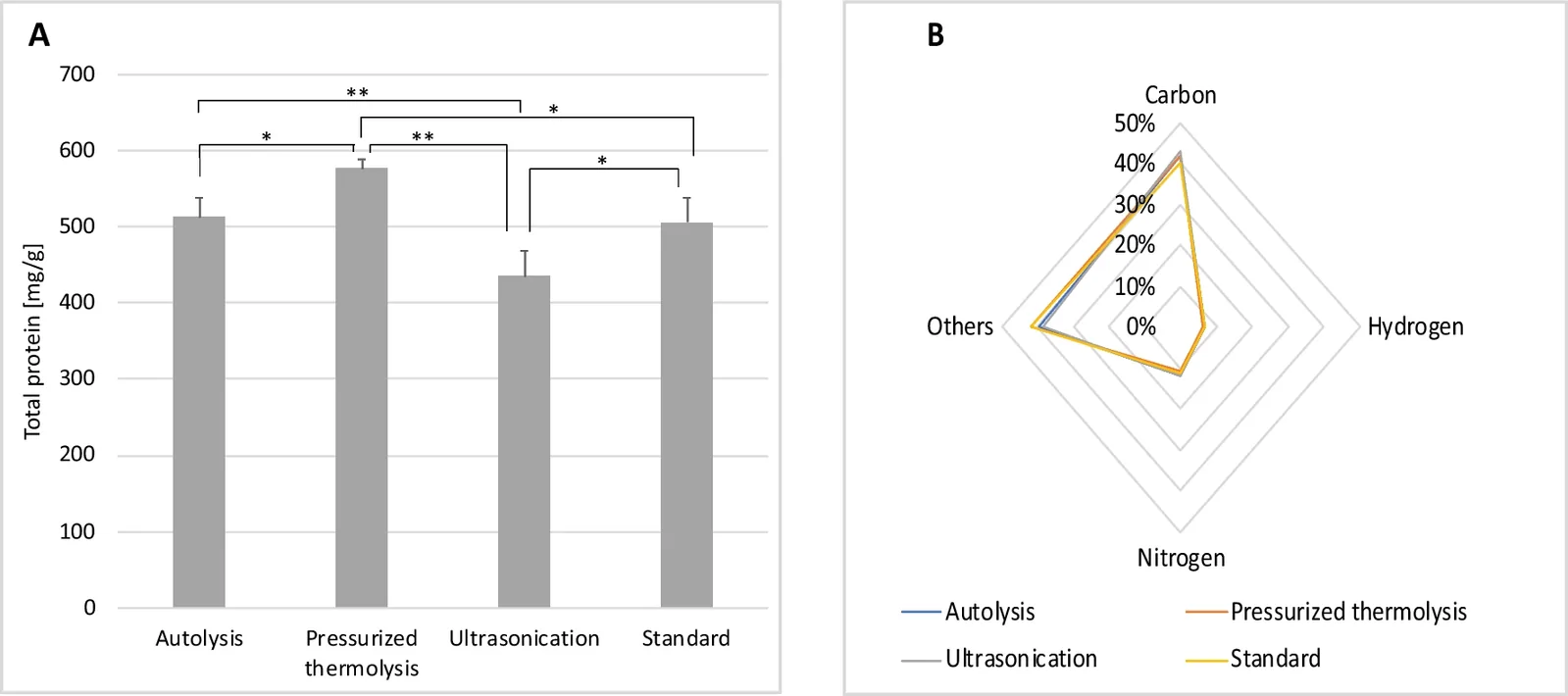
Compositional characteristics of yeast extracts. The protein content (**A**) of each yeast extract was estimated using Lowry’s colorimetric assay. The proportion of carbon, nitrogen and hydrogen, in the yeast extracts produced, was also quantified (**B**). Commercially sourced yeast extract (standard) was used as a control for comparative purposes. Statistically significant differences are indicated by * (*p* < 0.05) and ** (*p* < 0.01)

On the other hand, the protein content of yeast extracts produced from the residual brewers’ yeast ranged between 43 and 58% of dry mass (Fig. 1). Compared to the commercial yeast extract used as a control, the protein content of yeast extract produced via pressurised thermolysis was significantly (*p* = 0.013) higher than that obtained via ultrasonication, which contained a significantly (*p* = 0.012) lower protein content. The protein content of the yeast extract obtained by autolysis was comparable to that of the commercial yeast extract with no statistically significant difference (*p* = 0.762). With regard to elemental composition, the carbon (C) and nitrogen (N) contents are comparable in all samples (Fig. 1) with only slight variations, between 0.02 and 2.87% for C and 0.37 and 1.58% for N. Contrary to the results of the protein content, the lowest nitrogen content was observed in the yeast extract produced via pressurised thermolysis. Also, almost 3% more C atoms are observed in yeast extract produced via ultrasonication. Overall, autolysis yielded the highest amount of dried yeast extract (5.63 g), which is more than twice that obtained from ultrasonication (1.99 g) and over three times that resulting from pressurised thermolysis (1.48 g). Autolysis seems to be a more efficient method to produce yeast extract; it is thus no surprise that it is used in the commercial production of yeast extract.

### Identifying the optimum amount of soy molasses for growth of *X. dendrorhous *and astaxanthin production

**Fig. 2:**
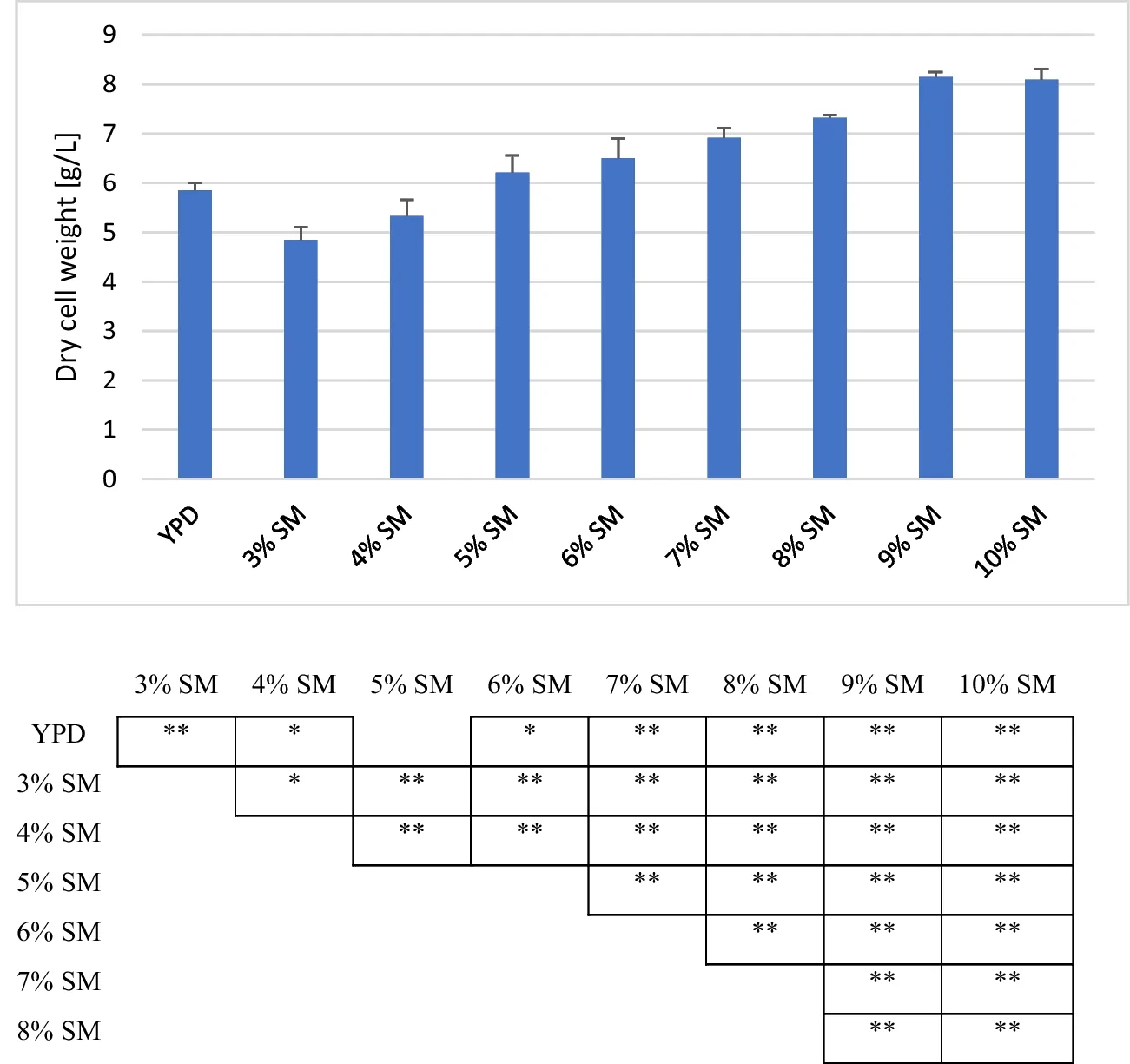
Biomass accumulation in media with different concentrations of soy molasses. *X. dendrorhous* strain Y1655 was cultivated in media containing different concentrations of soy molasses and 0.2% commercial yeast extract. The dry cell weight of cultures after 120 h of cultivation was estimated and compared with each other. Statistically significant differences are indicated by * (*p* < 0.05) and ** (*p* < 0.01)

Carbon substrate is an inevitable component of yeast fermentation; it provides both energy and building blocks for biomass accumulation as well as the production of desired metabolites. The type as well as the concentration of this substrate is critical to growth and productivity. Here, we showed that increasing the concentration of soy molasses in a cultivation medium results in a steady increase in biomass accumulation until an SM concentration of 9% (Fig. 2); increasing SM concentration further did not significantly affect the amount of biomass accumulated after 120 h of cultivation. At 5% SM, the amount of biomass accumulated at the end of the cultivation period was not significantly different (*p* = 0.151) from that obtained with the control cultivation medium (YPD). Below 5% SM, significantly less biomass accumulation was observed compared to YPD. On the other hand, SM concentrations above 5% yielded significantly higher biomass when compared to YPD. Overall, above 5% SM, statistically significant changes in biomass accumulation were observed only when the concentration of SM was increased by at least 2 percentage points. The highest biomass accumulation (8.1 g/L) was observed at 9% SM with the lowest at 3% SM (4.8 g/L).

**Fig. 3:**
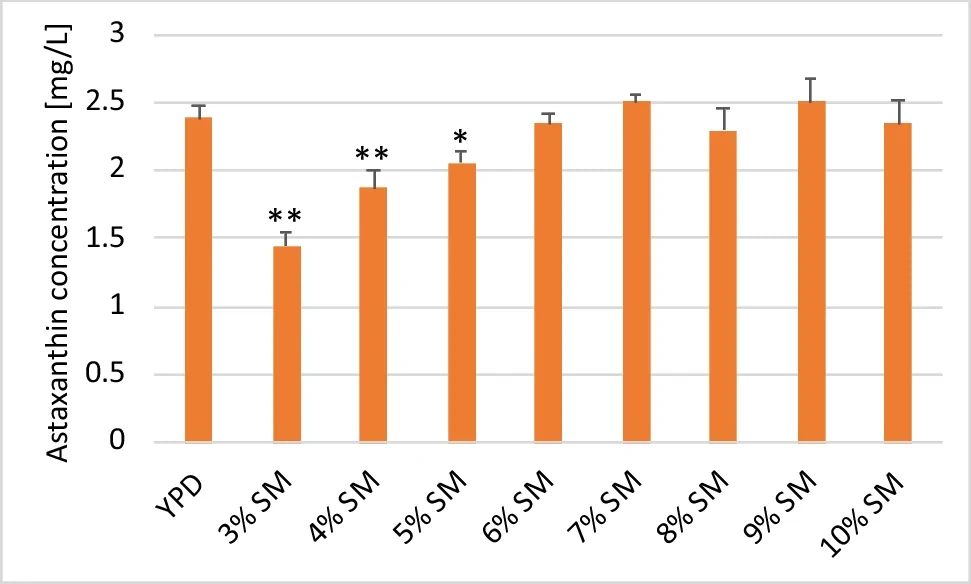
Astaxanthin production under different concentrations of soy molasses. Strain Y1655 was cultivated in media containing different concentrations of soy molasses and 0.2% commercial yeast extract. The amount of astaxanthin produced per litre of culture was estimated after 120 h of cultivation. Astaxanthin production on SM-based media was compared to that of YPD and statistically significant differences are indicated by * (*p* < 0.05) and ** (*p* < 0.01)

**Fig. 4:**
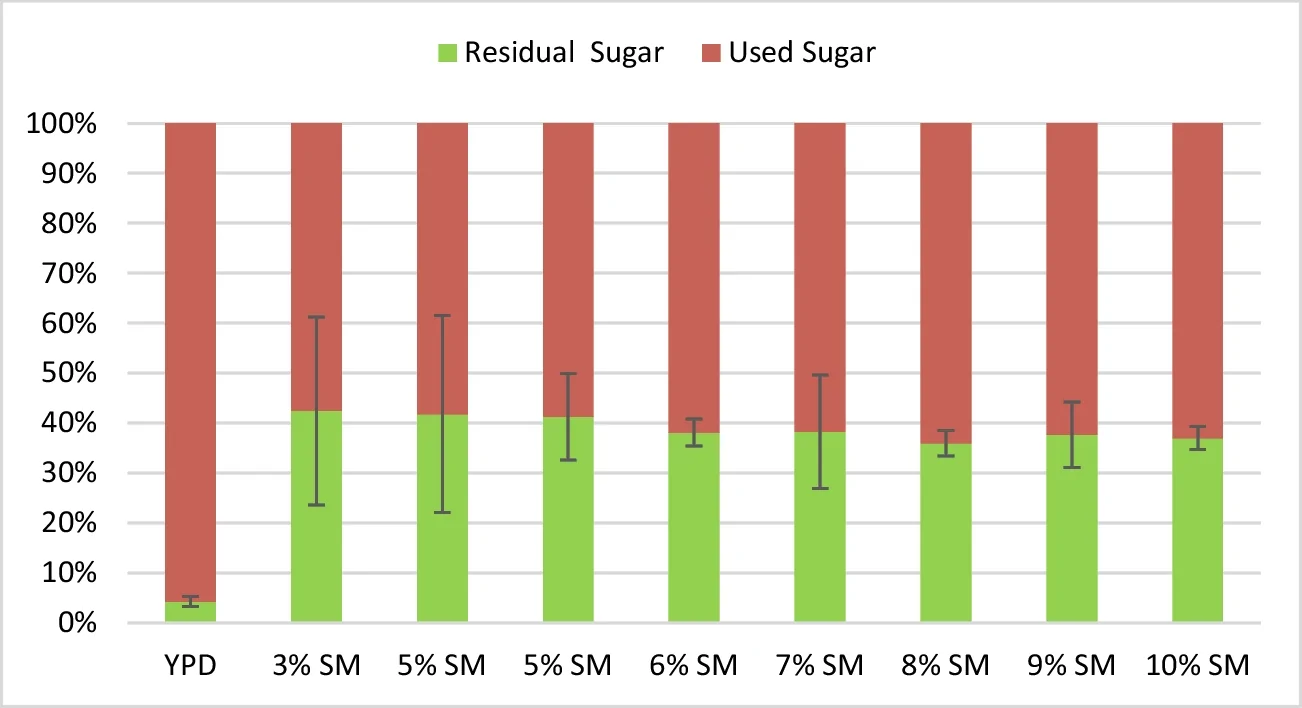
Total sugar consumption in media with different concentrations of soy molasses. Strain Y1655 was cultivated in media containing different concentrations of soy molasses and 0.2% commercial yeast extract. The total residual sugar (green) in the culture broth after 120 h of cultivation was quantified. The total consumed sugar (red) was estimated as the difference between the total initial sugar and the residual sugar

The effect of the starting sugar concentration on metabolite production is not always correlated with biomass accumulation and product formation. As such, it is important to optimise the initial sugar concentration of the media to allow for sufficient biomass and astaxanthin production. Accordingly, we evaluated the effect of different concentrations of soy molasses on astaxanthin production in *X. dendrorhous* strain Y1655. Similar to the trend observed with regard to dry cell weight (DCW), the volumetric astaxanthin production generally increased as the concentration of soy molasses in the media increased. The highest volumetric astaxanthin production was observed at 7% soy molasses concentration; no substantial changes in astaxanthin production were observed by increasing the amount of soy molasses beyond this concentration (Fig. 3). Notably, the concentration of astaxanthin observed in cultures cultivated on soy molasses at 6% and above was comparable to that of the control medium (YPD), with no statistically significant differences (*p* > 0.4). Although, at 6% soy molasses concentration, the total sugar in the cultivation medium was 21 g/L which is only slightly higher than that of YPD (20 g/L), it is worth noting that more than 35% of this sugar was not utilised (Fig. 4) which makes the amount of astaxanthin produced on such a medium more remarkable. As in the case of DCW, the lowest volumetric astaxanthin production was observed at 3% SM concentration.

Although holistically it appears that increasing the concentration of soy molasses corresponds with increased biomass and astaxanthin accumulation, to truly understand the effect of the initial sugar concentration on biomass and astaxanthin accumulation, it is necessary to estimate the total sugar utilised in each case. In the present study, the total residual sugar was estimated after 120 h of cultivation. At the end of the 120 h of cultivation, over 95% of the initial sugar present in YPD media was consumed by the yeast (Fig. 4). In contrast, between 35 and 41% of the initial sugar in SM-based media was not utilised by the yeast. Irrespective of the initial sugar concentration in SM-based media, the fraction of unassimilated sugar remains comparably the same.

Overall, the accumulation of astaxanthin in biomass was highest in the control media (YPD). Among the SM-based media, the highest astaxanthin accumulation in biomass was observed in 6% SM and 7% SM, which was significantly lower (*p* = 0.04) when compared to that of YPD (Table 1). Above 7% soy molasses concentration, the content of astaxanthin in the biomass tended to decrease as the concentration of soy molasses increased. No obvious trend was observed below 6% soy molasses concentration. The lowest astaxanthin accumulation in the biomass was observed in 3% SM-based medium. This notwithstanding, the highest amount of biomass produced per unit of SM was observed in 3% SM (161.56 mg/g of SM). Also, at 3% SM, about 20% to as high as 100% more astaxanthin was produced per unit of SM compared to other SM-based media. In general, the yield of both biomass and astaxanthin per unit of SM tended to decrease as the concentration of soy molasses in the growth media increased, with 10% SM resulting in the lowest levels (Table 1). Although no statistically significant difference was observed in terms of the amount of biomass accumulated at the endpoint (120 h) in 5% SM and 6% SM, significantly higher biomass was produced in 5% SM per unit of soy molasses compared to 6% SM (*p* = 0.003). In addition, similar amounts of astaxanthin were produced per unit of SM in both 5% SM and 6% SM, with no statistically significant difference observed (*p* = 0.364).

**Table 1:** Specific productivity of *X. dendrorhous* Y1655 cultivated in media with different concentrations of soy molasses

Media	Quantity of soy molasses (g/L)	Initial sugar (g/L)	Residual sugar (g/L)	Astaxanthin content of biomass (mg/g)	Biomass yield (mg/g of SM)	Astaxanthin yield (µg/g of SM)
YPD	NA	20.0	0.84	0.41	292.50^#^	119.28^#^
3% SM	30	10.5	4.45	0.30^**^	161.56	48.01
4% SM	40	14.0	5.85	0.35^*^	133.17	46.69
5% SM	50	17.5	7.21	0.33^*^	124.13	41.07
6% SM	60	21.0	7.99	0.36^*^	108.33	39.12
7% SM	70	24.5	9.37	0.36^*^	98.76	35.69
8% SM	80	28.0	10.08	0.31^**^	91.50	28.63
9% SM	90	31.5	11.86	0.31^**^	90.52	27.78
10% SM	100	35.0	12.93	0.29^**^	80.87	23.39

^*^Statistically significant at *p* = 0.05 when compared to YPD. ^**^Statistically significant at *p* = 0.01 when compared to YPD

^#^Per gram of glucose. *NA* not applicable

Although at 6% SM the volumetric astaxanthin output and total biomass accumulation are comparable to the control media, the specific yields of biomass and astaxanthin are reduced when compared to 5% SM. Besides, the final biomass concentration at 5% SM was similar to that of the control. Given these results, a 5% soy molasses concentration was selected for subsequent experiments.

### Effect of laboratory-produced yeast extract on growth of *X. dendrorhous *and astaxanthin production

**Fig. 5:**
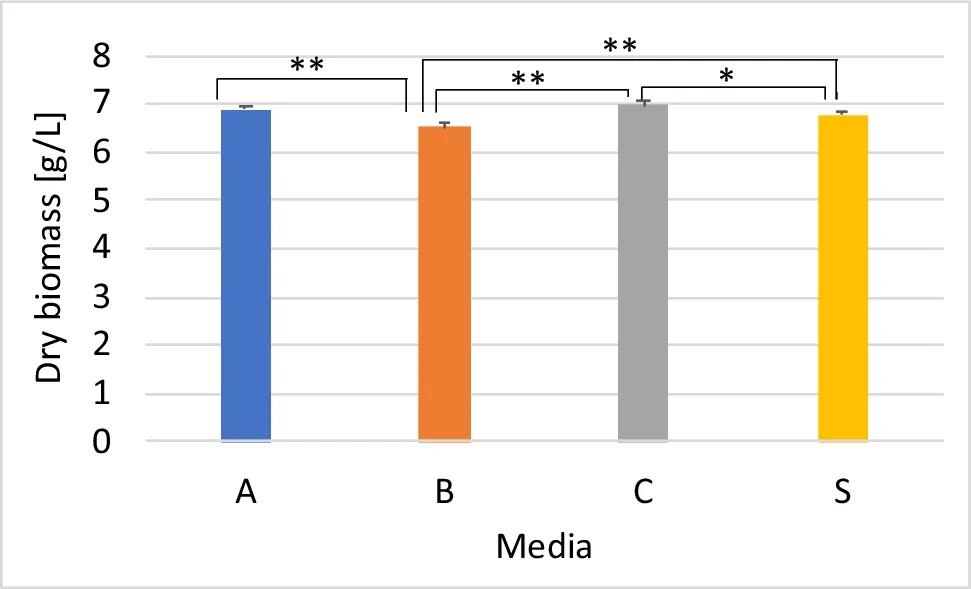
Biomass accumulation in media with different yeast extracts. Strain Y1655 was cultivated in media composed of 5% soy molasses and yeast extract (0.2%) obtained from A, autolysis; B, pressurised thermolysis; C, ultrasonication; and S, commercial yeast extract. The dry cell weight of cultures after 120 h of cultivation was estimated and compared with each other. Statistically significant differences are indicated by * (*p* < 0.05) and ** (*p* < 0.01)

To evaluate the laboratory-produced yeast extracts made from residual brewers’ yeast, media consisting of 5% soy molasses were supplemented, separately, with each of the yeast extracts produced. SM-based media with commercial yeast extract were used as the control. In general, a comparable final biomass concentration was obtained for all sample groups (Fig. 5), with slightly higher dry biomass weight observed for groups with the laboratory-produced yeast extracts in most cases. Strangely, even though the yeast extract obtained via pressurised thermolysis had the highest protein content (Fig. 1), the concentration of biomass of cultures cultivated on SM-based media containing yeast extract obtained via pressurised thermolysis (B) was significantly lower than the rest. Considering that all yeast extracts were subsequently sterilised at an even higher temperature and for a longer duration compared to that used in pressurised thermolysis, it is unlikely that the low biomass production observed was due to the thermal degradation of essential nutrients. Equally puzzling is the fact that the highest biomass accumulation was observed in media containing yeast extract obtained via ultrasonication (C), considering that the protein content of this yeast extract was the lowest. No significant difference (*p* = 0.901) was observed in the amount of biomass accumulated in media with commercial yeast extract or that containing yeast extract produced by autolysis, albeit with slightly higher biomass in the latter. Thus, the yeast extracts produced from residual brewers’ yeast can support the growth of the yeast to a similar extent as commercially available yeast extract.

**Fig. 6:**
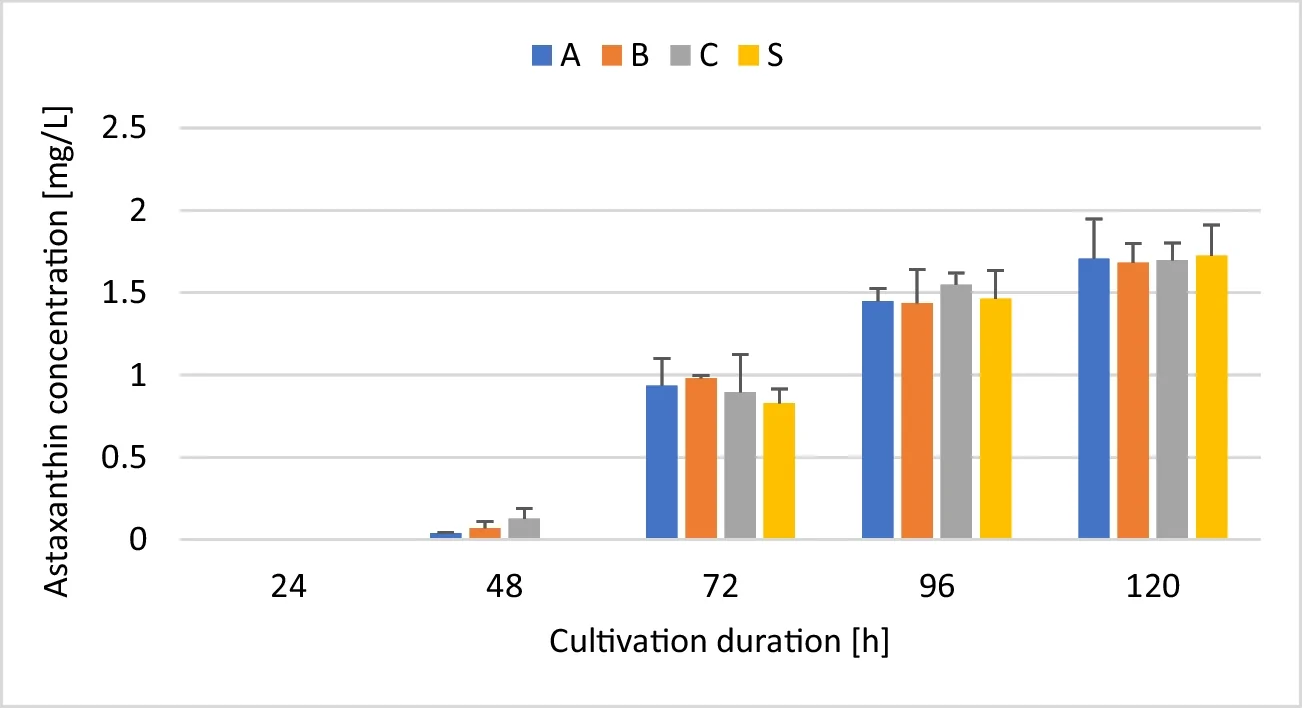
Volumetric astaxanthin output in SM-based media containing different yeast extracts. *X. dendrorhous*, strain Y1655, was cultivated in media consisting of 5% soy molasses and 0.2% yeast extract obtained from A, autolysis; B, pressurised thermolysis; C, ultrasonication; and S, commercial yeast extract. Samples were taken every 24 h to monitor astaxanthin production

Similar to DCW, no remarkable difference was observed regarding astaxanthin concentration at the end of the cultivation period (120 h). Except for slight variations, the accumulation of astaxanthin over the course of the study followed a similar trend (Fig. 6). Astaxanthin accumulation increased steadily throughout the study. The highest accumulation of astaxanthin within a 24-h window occurred between 48 and 72 h. Except for cultures in media C (< tenfold increase), an over tenfold increase in astaxanthin production was observed between 48 and 72 h. A similar pattern was observed when the yeast strain was cultivated using YPD under the same cultivation conditions (data not shown). Generally, astaxanthin accumulation is associated with the stationary phase which typically occurs within 48 h. However, cultivation of the yeast at 12 ℃ generally results in a prolonged lag phase during the first 24 h with the early stationary phase setting in around 72 h (data earmarked for a separate manuscript). As such, it is not surprising that the level of astaxanthin after 24 h was undetectable. All in all, astaxanthin production was not negatively affected by the yeast extracts produced. This is further visible in the cellular astaxanthin content as well as the amount of astaxanthin produced per unit of sugar consumed (Table S1 [Supplementary information]).

**Fig. 7:**
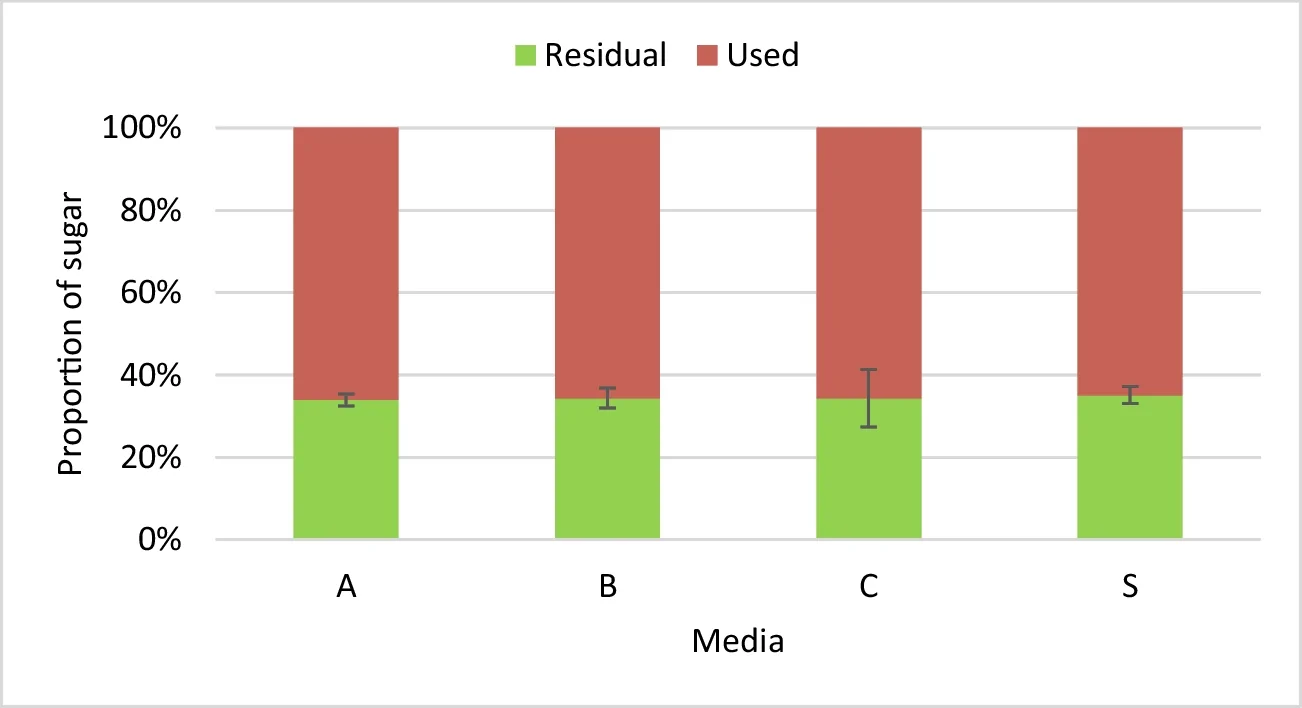
Effect of different yeast extracts on total sugar consumption. *X. dendrorhous*, strain Y1655, was cultivated in media consisting of 5% soy molasses and 0.2% yeast extract obtained from A, autolysis; B, pressurised thermolysis; C, ultrasonication; and S, commercial yeast extract. The total residual sugar in the culture broth after 120 h of cultivation was quantified. The initial total sugar (17.5 g/L) is represented as 100%

Predictably, the laboratory-produced yeast extracts did not affect the consumption of sugar by the yeast (Fig. 7), given that the elemental analysis showed the yeast extracts were characteristically similar to the commercial yeast extract. No significant difference was observed in the concentration of residual sugar across the different groups. The inability of the yeast to consume all the sugars in soy molasses is consistent with the results of the earlier section (Fig. 4). Also, the efficiency of conversion of sugar to either biomass or astaxanthin was not affected. Comparable specific yields were observed in all groups (Table S1 [Supplementary information]).

### Validation of two-component media for cultivation of *X. dendrorhous *and astaxanthin biosynthesis

**Fig. 8:**
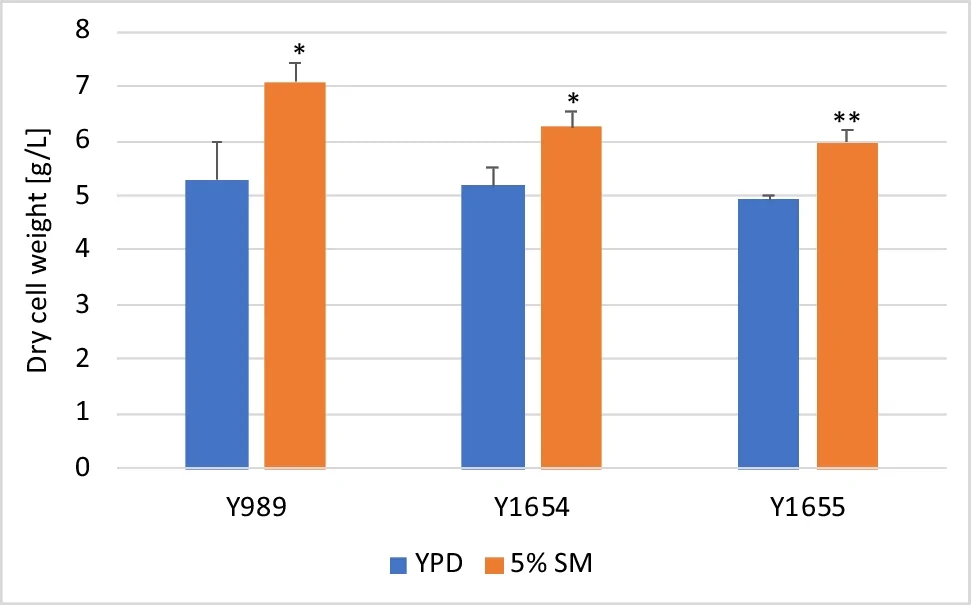
Biomass accumulation by different strains in molasses-based media. Growth of *X. dendrorhous* strain Y989, Y1654 and Y1655 were evaluated in the two-component medium composed of 5% soy molasses and 0.2% yeast extract obtained from autolysis. The dry cell weight of the cultures after 120 h of cultivation was estimated. For each strain, the growth in the SM-based medium was compared to growth in YPD, and statistically significant differences are indicated by * (*p* < 0.05) and ** (*p* < 0.01)

**Fig. 9:**
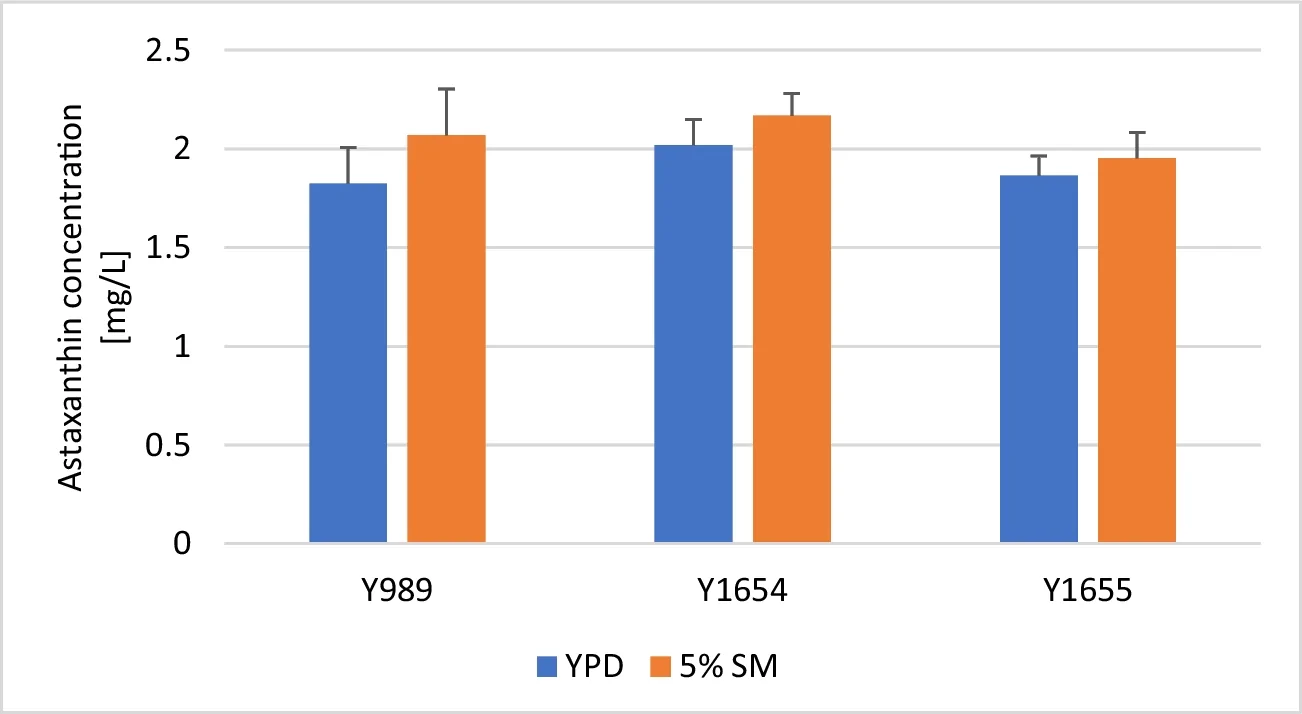
Volumetric astaxanthin output of different strains in molasses-based media. *X. dendrorhous* strains Y989, Y1654 and Y1655 were cultivated, independently, for 120 h in the two-component growth medium, composed of 5% soy molasses and 0.2% yeast extract obtained from autolysis. The astaxanthin production in the two-component SM-based medium was assessed and compared to that of YPD for each strain

The capacity of the growth medium formulated in this study to support biomass and astaxanthin accumulation by the yeast was further corroborated with different strains of the yeast. To this end, biomass accumulation and astaxanthin production of three yeast strains were evaluated on the formulated medium and compared to YPD. Interestingly, the SM-based medium (consisting of 5% soy molasses and 0.2% yeast extract obtained via autolysis of residual brewers’ yeast) resulted in at least 20% more DCW than YPD, in all strains (Fig. 8). In terms of astaxanthin production, between 4 and 13% more astaxanthin was achieved with SM-based medium depending on the strain (Fig. 9). Overall, the SM-based medium resulted in higher astaxanthin production by the yeast after 120 h of cultivation. However, the differences were not statistically significant compared to YPD. This notwithstanding, the cellular astaxanthin content was generally higher when cells were cultivated in YPD compared to an SM-based medium (Table S2 [Supplementary information]). There seems to be a trade-off between biomass production and the astaxanthin content of the biomass in an SM-based growth medium. This trend was also visible in the earlier section during the evaluation of different soy molasses concentrations. This is understandable given that both biomass and astaxanthin production rely on common central carbon metabolism metabolites. Holistically, the reduced cellular astaxanthin content was, however, compensated by the increased accumulation of astaxanthin-containing biomass in the SM-based media. Furthermore, the concentration of residual sugar in YPD and SM-based growth medium was, respectively, similar across the three strains. Over 96% of the initial sugar in YPD was assimilated at the end of the study by each strain. On the other hand, only about 65% of the initial sugar in the SM-based growth medium was consumed (Figure S2) [Supplementary information]). The residual sugars, as mentioned earlier, consist primarily of melibiose and manninotriose, arising from the partial hydrolysis of raffinose and stachyose (Figure S4 [Supplementary Information]). This is consistent with earlier observations regarding strain Y1655 and as well our previous study regarding strain Y1654 (Kanwugu et al. 2020). With regard to specific yields, SM-based medium appears superior. Twice as much biomass and astaxanthin are produced per unit of sugar consumed in SM-based media compared to YPD (Table S2 [Supplementary information]).

## Discussion

Soy molasses (SM) is a by-product of soybean protein concentrate production; as such, its composition of sugar and other nutrients generally varies between batches and among manufacturers, in part, due to differences in the starting material and industrial processes leading up to its production. In the present study, the total sugar content of the molasses used was found to be around 35% w/w, consisting predominately of sucrose, raffinose and stachyose, with a substantial fraction of monosaccharides also present. In general, it is reported that SM contains at least 30% w/w carbohydrates which are comprised largely of mono- and disaccharides (sucrose, ≤ 50%; fructose, < 8%, glucose, < 8%) and a substantial amount of raffinose-family oligosaccharides (raffinose, < 20%; stachyose, ≥ 25%) which are not fermentable by yeast (Rodrigues et al. 2021; Romão et al. 2012; Wang et al. 2019). One of the major advantages *X. dendrorhous* has over other microbial astaxanthin producers is that it is able to utilise an array of carbon sources for growth including glucose, fructose and sucrose (An et al. 2001) which together constitute over half the total carbohydrate in molasses making it a suitable carbon substrate. Furthermore, previous studies indicate that soy molasses contains up to 9.4% w/w proteins (Siqueira et al. 2008). Elemental analysis of the soy molasses used in the present studies evidently indicates the presence of nitrogenous compounds. SM is also known to contain lipids and phenolic compounds predominantly isoflavones (Duru et al. 2022). In addition, SM contains an appreciable amount of mineral ions including calcium, cobalt, iron, magnesium, manganese, phosphorus, sodium and sulphite which supports the growth and metabolism of yeast (Rodrigues et al. 2021). Together, this makes SM an attractive substrate in biotechnology.

Yeast extract, an autolysate composed of soluble yeast components, is a widely used nutritional resource in microbial cultivation. It provides essential nutrients such as vitamins, trace elements and growth factors for the normal growth of yeast cells. The most commonly used method for the production of commercial yeast extract is autolysis (Tomé 2021). As such, it is not surprising that the protein content of the yeast extract obtained by autolysis in the present study was comparable to that of the commercial yeast extract with no statistically significant difference (*p* = 0.762). Despite significant variations in the protein content of the yeast extracts produced in the present study, elemental analysis revealed a similar proportion of carbon (C) and nitrogen (N) respectively across the board. Nonetheless, slightly more carbon atoms were observed in the yeast extract produced through ultrasonication. These extra carbon atoms might be originating from sugar molecules released from the cell wall. In fact, de Carvalho Silvello et al. observed that ultrasonication enhances the enzymatic release of reducing sugar in sugarcane bagasse (de Carvalho Silvello et al. 2019); thus, it is possible that the ultrasound treatment promoted the activity of native hydrolytic enzymes resulting in the release of more sugar molecules from the cell wall.

For the cultivation of *X. dendrorhous*, simple sugars are commonly used as the carbon and energy source, particularly glucose. Although, in general, a higher concentration of carbon source translates into higher and faster biomass accumulation, increasing the concentration beyond a certain threshold affects growth negatively. This is usually due to pH or osmolarity changes or in some cases the wasteful utilisation of glucose via incomplete oxidation (i.e. the Crabtree effect) and rapid accumulation of toxic wastes such as ethanol. *X. dendrorhous* is a Crabtree-positive yeast; as such, at sufficiently high glucose concentrations and inadequate oxygen concentration, overflow metabolism is activated, which could lead to decreased biomass accumulation (Vega-Ramon et al. 2021). Generally, a glucose concentration around 20 g/L is optimum for the cultivation of *X. dendrorhous*. At glucose concentrations ≥ 40 g/L, biomass yield decreases significantly (Miao et al. 2019; Yamane et al. 1997). No decreased biomass accumulation was observed in the current study when the concentration of the carbon source, i.e. SM in the media, was increased up to 10% w/v corresponding to a total sugar concentration of 35 g/L. However, increasing the concentration of SM in media beyond 9% did not result in a corresponding increase in biomass accumulation. While Ramírez et al. (2001) previously reported that biomass formation increased as a function of carbon concentration while using date juice as a carbon source, it has also been reported that relatively more biomass is obtained at a lower C/N ratio (Vustin et al. 2004). Compared to YPD (10:1), a higher C/N ratio (13:1 to 15:1) was observed in SM-based media. This notwithstanding, SM contains substantial amounts of micronutrients which are beneficial for yeast growth (Rodrigues et al. 2021). Moreover, other studies have observed that the final biomass concentration increases with increasing C/N ratio (Pan et al. 2017). Also, compared to glucose, cultivation of different strains of *X. dendrorhous* on sucrose-based media results in increased biomass accumulation and higher specific biomass yield (Vázquez et al. 1997). In this regard, it is worth noting that sucrose is the major sugar in the soy molasses utilised.

Aside from biomass accumulation, the concentration of sugar in the starting medium could have a considerable effect on the biosynthesis of metabolites. The carbon to nitrogen ratio is reported to be a crucial factor affecting microbial carotenoid production. Whereas high nitrogen content may result in the downregulation of secondary metabolite synthesis, nitrogen limitation could trigger the increased accumulation of fatty acids instead (Stoklosa et al. 2019). Previous studies have established that higher C/N ratios promote astaxanthin accumulation (Pan et al. 2017; Yamane et al. 1997). In the present study, the C/N ratio was generally higher in SM-based media compared to YPD, with slight increments as SM concentration increases. Among the SM-based media, while 3% SM media had the lowest C/N ratio (13:1), the C/N ratios at 6% SM and above were similar (approx. 15:1). Although the exact molecular mechanism mediating how C/N ratios affect astaxanthin production in the yeast is not known, Pan et al. observed that isopentenyl pyrophosphate isomerase and astaxanthin synthase (enzymes directly involved in the astaxanthin biosynthesis pathway) were upregulated as the C/N ratio increased (Pan et al. 2017). Nevertheless, no obvious trend was observed regarding the C/N ratio and the final astaxanthin concentration.

It is widely known that different carbon sources are assimilated and metabolised to varying extents by *X. dendrorhous*. For instance, at an initial total sugar concentration of 20 g/L, while glucose consumption is completed by 72 h, it takes an extra 24 h for arabinose consumption to be complete, and for xylose, consumption was not complete even after 168 h (Stoklosa et al. 2019). In the present study, while almost all the sugar in the control media (YPD) is always consumed by 120 h, a significant portion of the sugar present in SM-based media always remains at the cultivation endpoint (120 h). This unassimilated fraction consists mainly of melibiose and manninotriose. These sugars result from the hydrolysis of the β-1,2 glycosidic bonds between glucose and fructose in raffinose and stachyose, respectively, by the action of invertase (β-fructosidase), as depicted in Figure S5 (Supplementary Information). The accumulation of melibiose and manninotriose, which contain α-1,6 glycosidic bonds, suggests the yeast’s inability to metabolise these products further due to the lack of the prerequisite enzyme, α-galactosidase. The inability of the yeast to completely utilise the sugars present in the soy molasses is unsurprising, as similar observations were made in our previous study when strain Y1654 was grown on soy molasses-based media (Kanwugu et al. 2020). Consistent with the observation of the present study, an earlier report around the time of the discovery of the yeast noted that the yeast is able to weakly metabolise raffinose (Miller et al. 1976). In addition, moderate raffinose metabolism has been reported in an NTG mutant strain of the yeast, which even resulted in higher astaxanthin accumulation in cells compared to glucose, sucrose or fructose (Fang and Cheng 1993). This ability, perhaps, is strain specific.

For the biotechnological production of astaxanthin to successfully compete with chemical synthesis, the cost of production must be greatly reduced. In this regard, the cost of media components for the cultivation of microorganisms to produce astaxanthin can be reduced by developing new growth media that utilises inexpensive raw materials. In the present study, a two-component cultivation medium was formulated using soy molasses (SM) and yeast extract obtained from residual brewers’ yeast. A significant portion of the sugar in soy molasses is not assimilable by *X. dendrorhous*. Irrespective of this, SM-based media containing at least 5% soy molasses supports the growth of *X. dendrorhous* to levels similar to or greater than that observed with standard glucose-based media (YPD). Furthermore, astaxanthin production on such media is comparable to that achieved with YPD, although the cellular content of astaxanthin is lower when cells are cultivated on SM-based media. The cellular content of astaxanthin in SM-based media could, however, be augmented by using superior astaxanthin-producing strains. While increasing the concentration of soy molasses beyond 5% results in increased biomass accumulation and astaxanthin titre, at least up to some point, it nonetheless appears to be a wasteful approach with the strains tested as the efficiency of conversion of sugar to biomass or product is greatly reduced. The yeast extract produced from residual brewers’ yeast was characteristically similar to the commercial variant and supported the growth of *X. dendrorhous* as well as astaxanthin production just as much. To further improve the sustainable utilisation of feedstock, the complex sugars can be hydrolysed to simple sugars with greener techniques such an enzyme hydrolysis prior to reconstitution into the growth medium. Besides, *X. dendrorhous* could as well be equipped with the prerequisite enzymes via strain engineering to metabolise the complex sugars during cultivation. However, engineering tools and techniques for *X. dendrorhous* are currently limited. Given that the growth medium formulated in this study utilises industrial wastes as feedstock, it provides an opportunity to reduce the production cost of astaxanthin from *X. dendrorhous* while simultaneously reducing the environmental impact related to the disposal of such industrial wastes.

## Supplementary Information

Below is the link to the electronic supplementary material.

Supplementary file1 (PDF 697 KB)

## Data Availability

All data supporting the findings of this study are available within the paper and its Supplementary Information.
