# Supplementary material for: Biovalorisation of agro-industrial wastes into astaxanthin by Xanthophyllomyces dendrorhous
Source: Appl Microbiol Biotechnol. 2024 Jul 27;108:429. doi: 10.1007/s00253-024-13257-5 (PMC11283421; doi:10.1007/s00253-024-13257-5)
Supplement: Supplementary file 1 [file 253_2024_13257_MOESM1_ESM.pdf]

Applied Microbiology and Biotechnology

Biovalorization of agro-industrial wastes into astaxanthin by *Xanthophyllomyces dendrorhous*

Osman N. Kanwugu[1,2 *], Ibrahim Ibn-Wuni[1], Vadim A. Shevyrin[1], Thomas C. Williams[2], Tatiana V. Glukhareva[1]

[1]*Institute of Chemical Engineering, Ural Federal University named after the first President of Russia B.N. Yeltsin, Mira street 28, 620002 Ekaterinburg, Russia.*

[2]*School of Natural Sciences, and ARC Centre of Excellence in Synthetic Biology, Macquarie University, Sydney, NSW 2109, Australia.*

*Correspondence: nabayire@gmail.com or osman.kanwugu@hdr.mq.edu.au

Supplementary information

Table S1. Specific productivity of *X. dendrorhous* Y1655 cultivated in molasses-based media with different yeast extract.

Media	Astaxanthin content of biomass (mg/g)	Biomass yield (mg/g of SM)	Astaxanthin yield (µg/g of SM)
A	0.25	137.73	34.13
B	0.26	130.27	33.63
C	0.24	139.07	33.93
S	0.25	135.60	34.49

Table S2. Specific productivity of *X. dendrorhous* cultivated in molasses-based media with yeast extract obtained from autolysis.

Strain	Media	Residual sugar (g/L)	Astaxanthin content of biomass (mg/g)	Biomass yield per sugar consumed (mg/g)	Astaxanthin yield per sugar consumed (mg/g)
Y989	YPD	0.76	0.35	274.82	0.09
	SM	6.13	0.29*	622.65**	0.18**
Y1654	YPD	0.68	0.39	268.46	0.10
	SM	6.38	0.35	561.40**	0.20**
Y1655	YPD	0.70	0.38	255.55	0.10
	SM	6.03	0.33	520.81*	0.17**

*statistically significant at p= 0.05, **statistically significant at p= 0.01 when compared to YPD

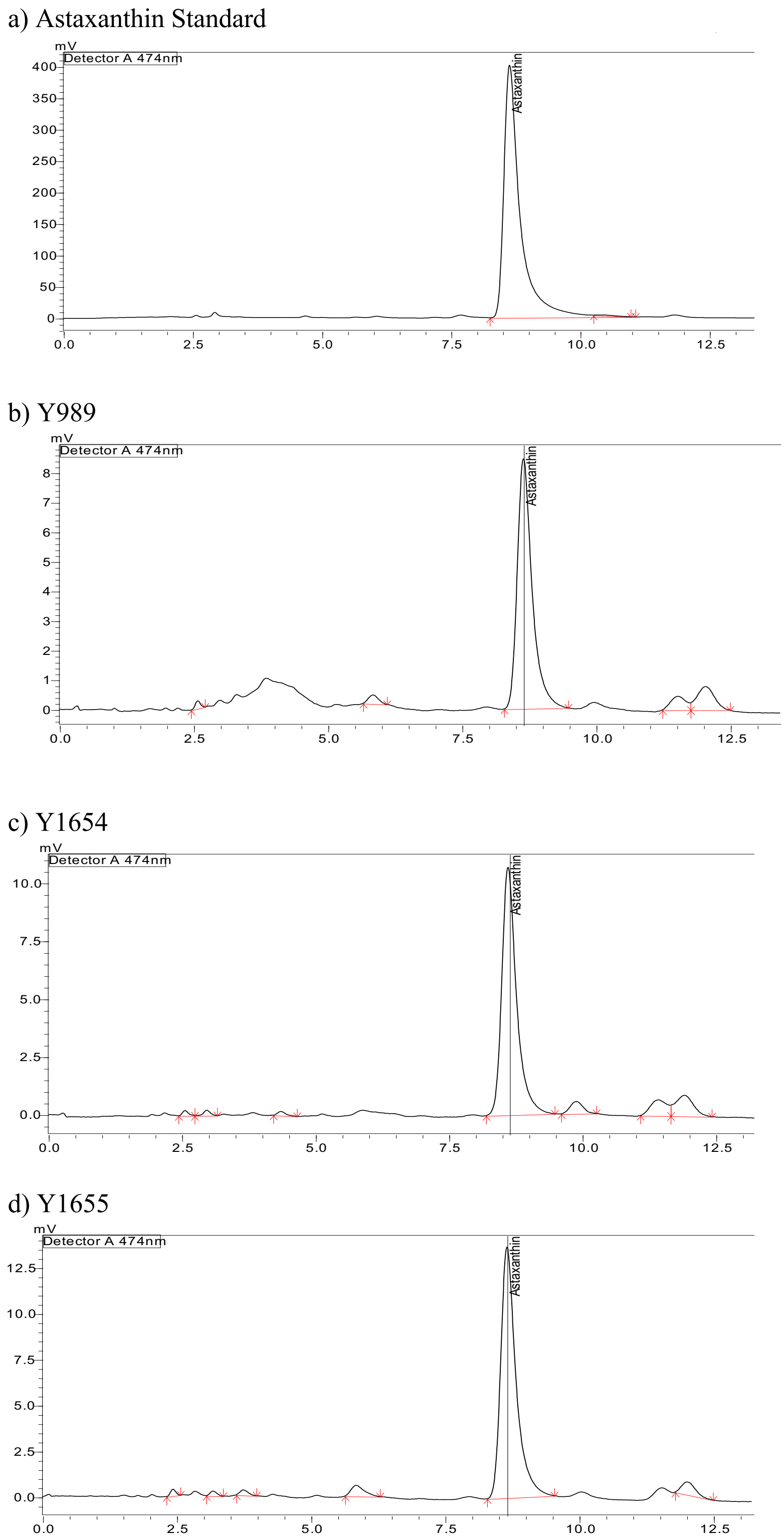

Figure S1

Typical chromatogram of carotenoid extract of *X. dendrorhous* cells cultivated in SM-based medium.

Strains of *X. dendrorhous* Y989, Y1654 and Y1655 were cultivated in 5% SM-based medium under continuous white illumination at 12 °C. Carotenoids were extracted from cells after 120 hours of cultivation and separated on an HPLC. Astaxanthin was identified on a UV/Vis detector at 474 nm based on the retention time of a chemically synthesised astaxanthin standard. The chromatograms have been truncated for ease of presentation.

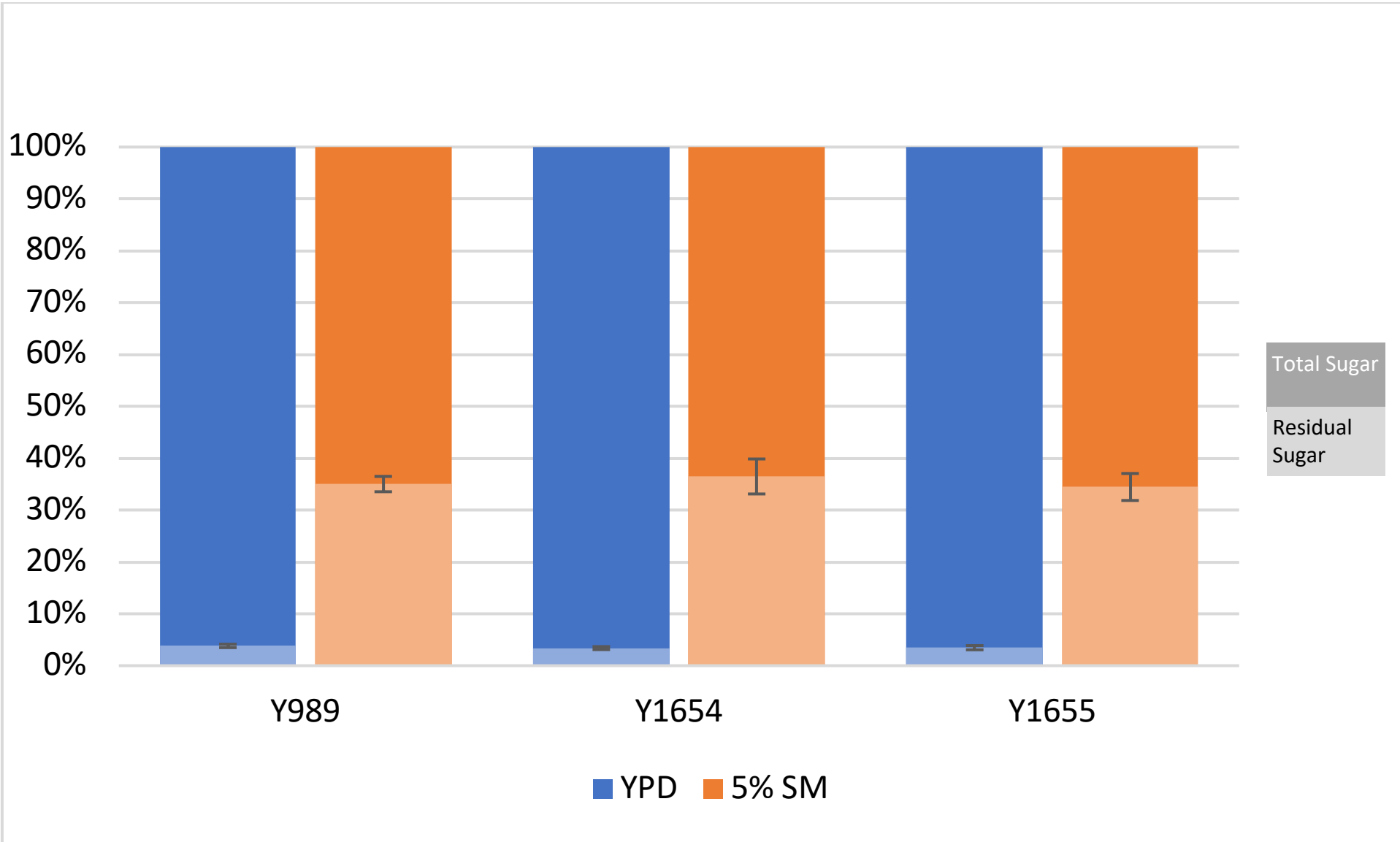

Figure S2

Total sugar consumption of different strains in molasses-based media.

All three strains (Y989, Y1654 and Y1655) were cultivated independently in the two-component SM-based media and YPD. The total residual sugar in the culture broth after 120 h of cultivation was quantified. The initial total sugar (17.5 g/L for SM-based media and 20 g/L for YPD) is represented as 100%

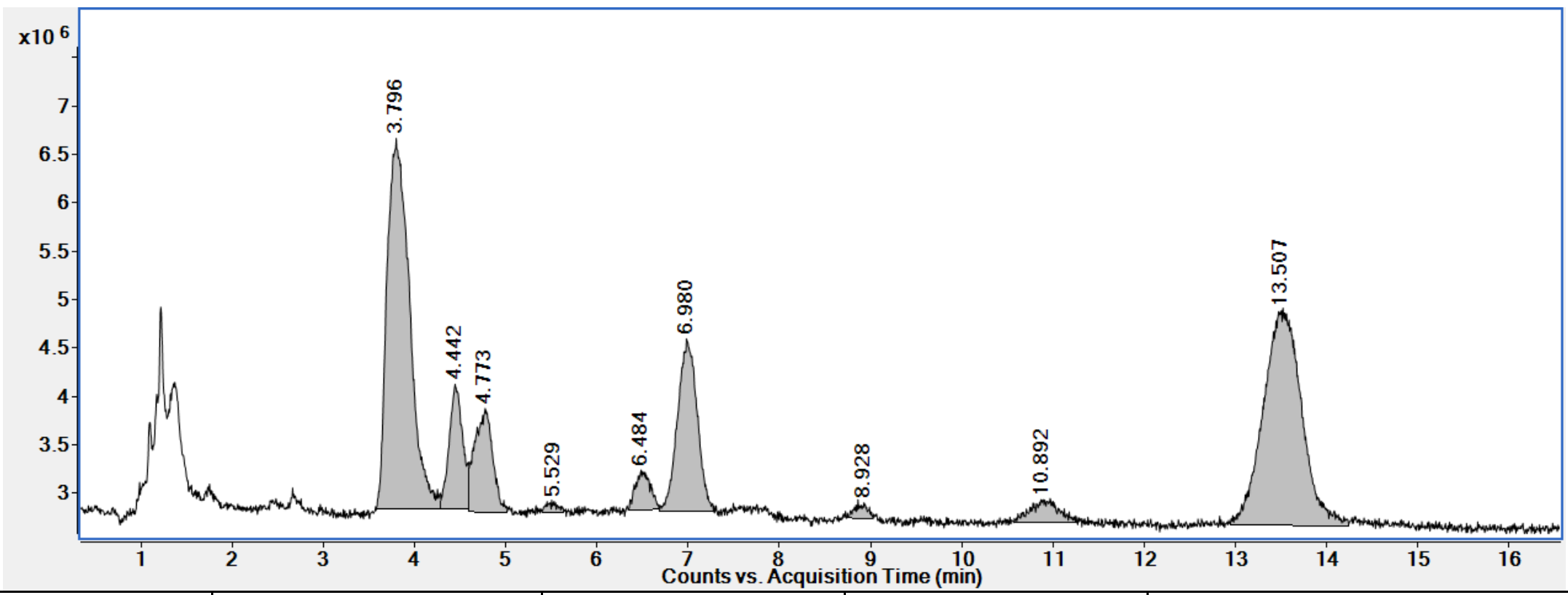

Retention time (t_R, min)	Accurate mass [M + Cl]$^-$	Exact mass [M + Cl]$^-$	Molecular formula	Tentative identification
3.8	377.0861	377.0856	$C_{12}H_{22}O_{11}$	Sucrose
4.4	391.1018	391.1013	$C_{13}H_{24}O_{11}$	methylated carbohydrate
4.8	391.1018	391.1013	$C_{13}H_{24}O_{11}$	methylated carbohydrate
5.5	377.0860	377.0856	$C_{12}H_{22}O_{11}$	Melibiose
6.5	377.0862	377.0856	$C_{12}H_{22}O_{11}$	-
7.0	539.1393	539.1384	$C_{18}H_{32}O_{16}$	Raffinose
8.9	553.1542	553.1541	$C_{19}H_{34}O_{16}$	-
10.9	539.1389	539.1384	$C_{18}H_{32}O_{16}$	Manninotriose
13.5	701.1920	701.1913	$C_{24}H_{42}O_{21}$	Stachyose

Figure S3

A total ion chromatogram depicting the saccharide composition of soy molasses.

a) Y989

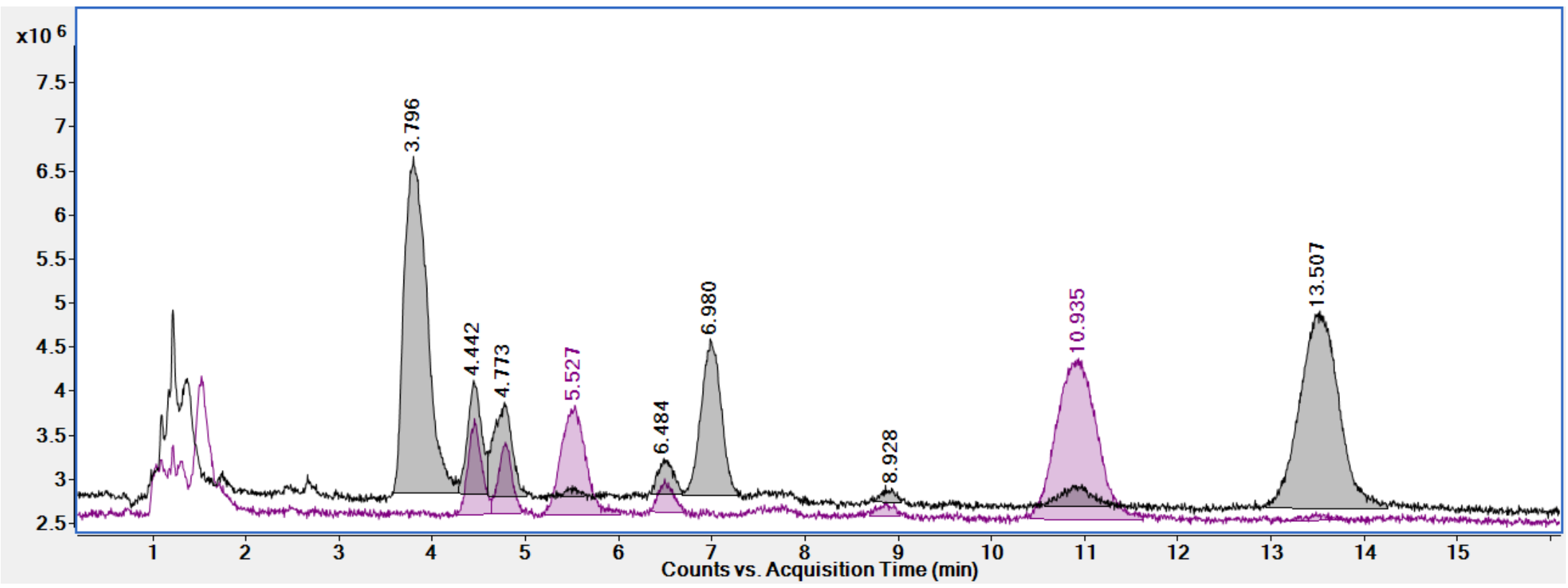

b) Y1654

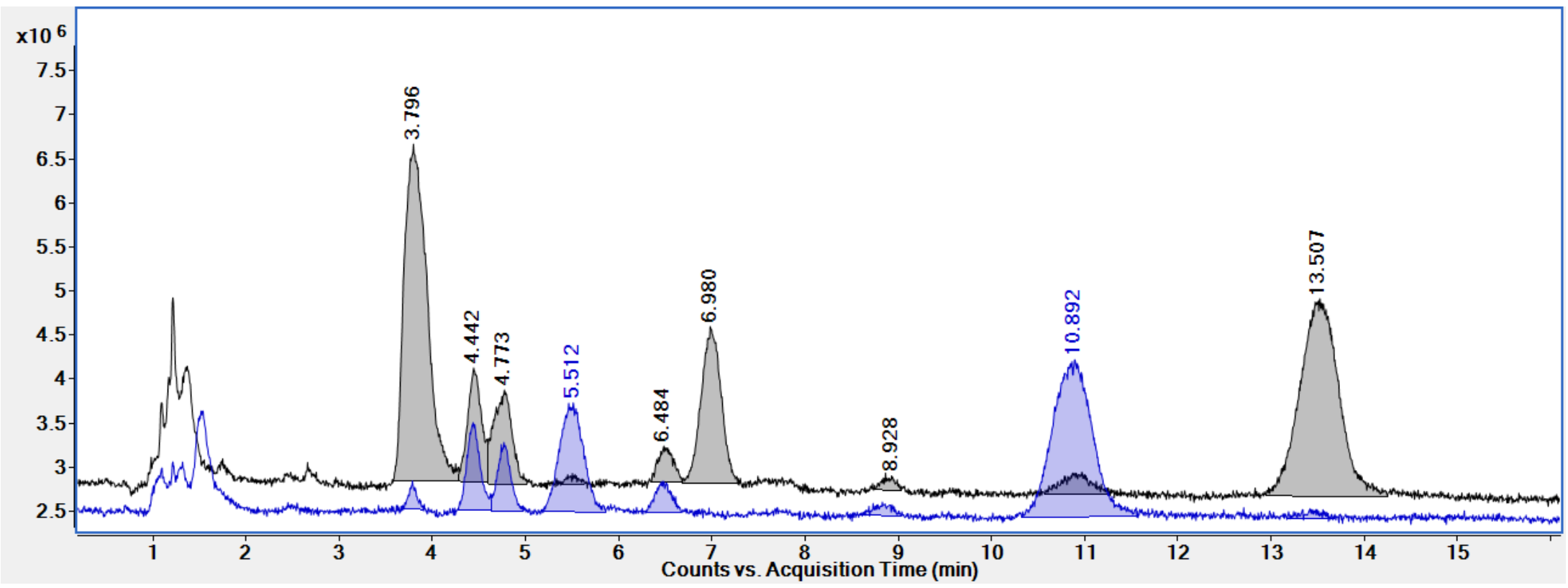

c) Y1655

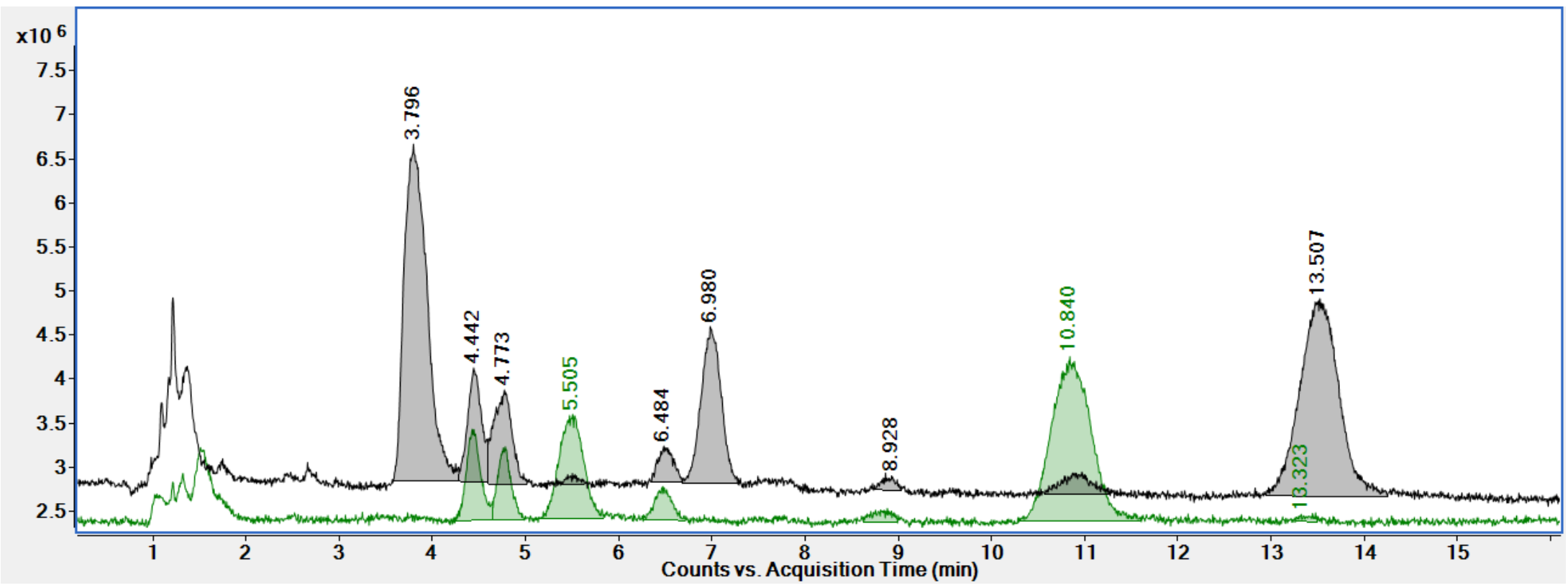

Retention time (t_R, min)	Accurate mass [M + Cl]$^-$	Exact mass [M + Cl]$^-$	Molecular formula	Tentative identification
3.8	377.0861	377.0856	$C_{12}H_{22}O_{11}$	Sucrose
4.4	391.1018	391.1013	$C_{13}H_{24}O_{11}$	methylated carbohydrate
4.8	391.1018	391.1013	$C_{13}H_{24}O_{11}$	methylated carbohydrate
5.5	377.0860	377.0856	$C_{12}H_{22}O_{11}$	Melibiose
6.5	377.0862	377.0856	$C_{12}H_{22}O_{11}$	-
7.0	539.1393	539.1384	$C_{18}H_{32}O_{16}$	Raffinose
8.9	553.1542	553.1541	$C_{19}H_{34}O_{16}$	-
10.9	539.1389	539.1384	$C_{18}H_{32}O_{16}$	Manninotriose
13.5	701.1920	701.1913	$C_{24}H_{42}O_{21}$	Stachyose

Figure S4

Total ion chromatograms of culture supernatant after 120 hours of cultivating strain Y989 (a); Y1654 (b); and Y1655 (c) in 5% SM-based growth medium. The chromatograms of culture supernatant (purple, violet, and green) are overlaid on top of the total ion chromatogram (gray) of soy molasses at 5% concentration.

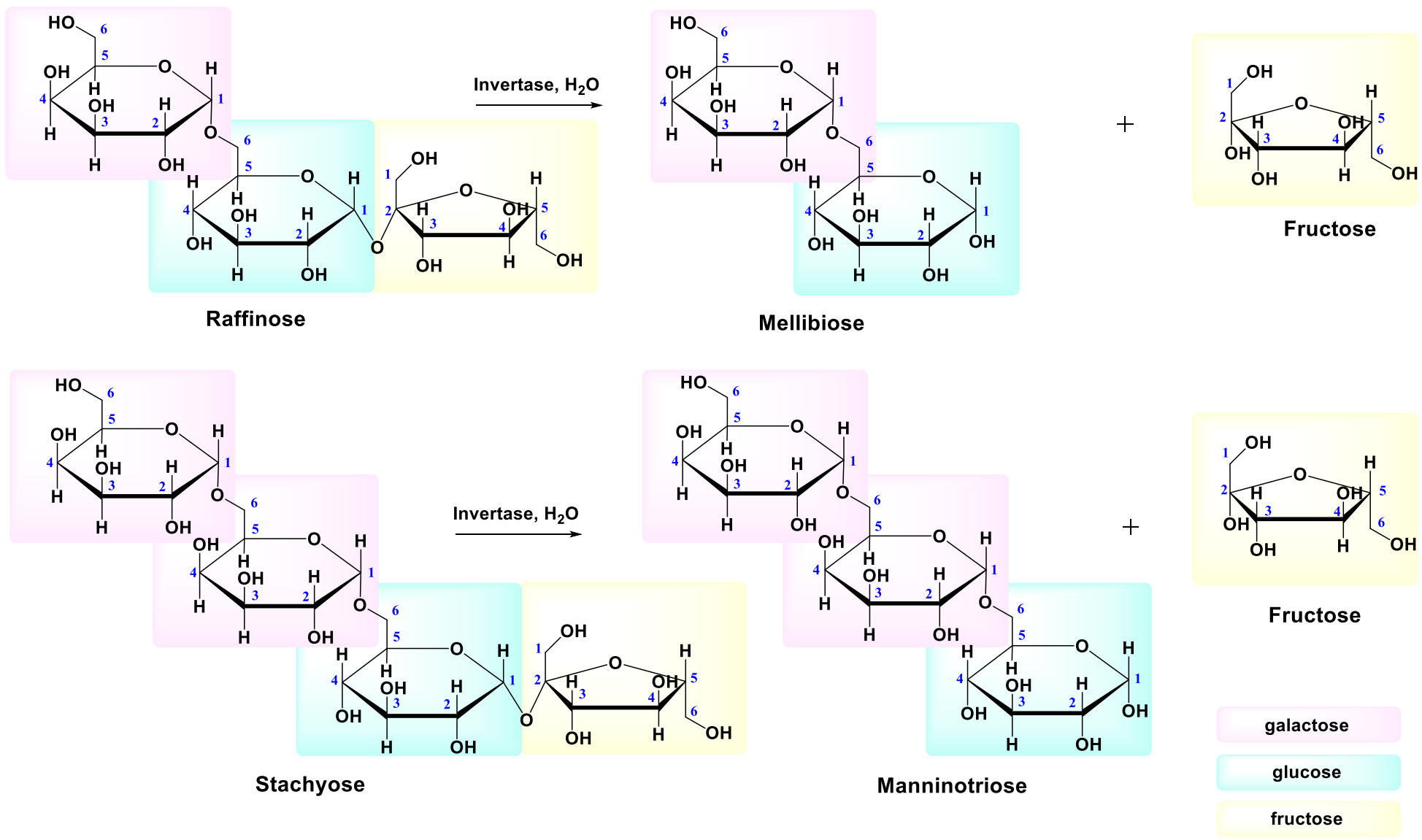

Figure S5

Partial hydrolysis of raffinose and stachyose by invertase ((β-fructosidase))
